# Informed consent for exome sequencing in diagnostics: exploring first experiences and views of professionals and patients

**DOI:** 10.1111/cge.12299

**Published:** 2013-11-04

**Authors:** T Rigter, CJA van Aart, MW Elting, Q Waisfisz, MC Cornel, L Henneman

**Affiliations:** a Department of Clinical Genetics, Section of Community Genetics, EMGO Institute for Health and Care Research, VU University Medical CenterAmsterdam, the Netherlands; b Department of Clinical Genetics, VU University Medical CenterAmsterdam, the Netherlands; c Centre for Medical Systems BiologyLeiden, the Netherlands; d CSG Centre for Society and the Life SciencesNijmegen, the Netherlands

**Keywords:** exome sequencing, high-throughput nucleotide sequencing, incidental findings, informed consent, molecular diagnostics, qualitative research, unsolicited findings

## Abstract

Next-generation sequencing is increasingly being chosen as a diagnostic tool for cases of expected genetic, but unresolved origin. The consequential increased need for decisions on disclosure of unsolicited findings poses a challenge for the informed consent procedure. This study explored the first experiences with, and needs for, the informed consent procedure in diagnostic exome sequencing, with the stakeholders involved. Semi-structured interviews were conducted with 11 professional experts and one professional gave a written response. Furthermore, the counseling process was observed in three cases where exome sequencing was offered, followed by interviews with the patient (representative) and the genetic counselor. The respondents not only preferred an opt-out for unsolicited findings but also identified many challenges and therefore more experiences with exome sequencing was considered needed. Context-dependent decision-making was observed and an Advisory Board for unsolicited findings was considered helpful while doubts were raised about the feasibility and the possibility of undermining patients' autonomy. Finally, respondents brought up the complexity of information provision, and division of responsibilities between clinicians and the lab. These challenges and needs, raised by stakeholders involved, provide more insight in the next steps needed for an optimal informed consent procedure for exome sequencing in diagnostics.

In recent years next-generation sequencing (NGS) and analyzing large parts of the genome have moved from research to the diagnosis of patients with expected genetic disorders with unresolved etiology 1–4. This approach, however, increases the chance of unsolicited (often referred to as incidental) findings, giving rise to multiple challenges 4–6.

Challenges lie for example in the disclosure of unsolicited findings to patients: which information is perceived relevant, what do patients expect and prefer 7, and how to effectively prepare patients for the potential receipt of unsolicited findings 8,9?

There is ongoing debate about the autonomy of patients and specifically about how to do justice to their right to know about their health information (e.g. as stressed by the American College of Medical Genetics), while safeguarding their right to refuse this information [as discussed by the European Society of Human Genetics (ESHG) and others] 7,10–13. Although several attempts have been made to give guidance on these themes 7,14–16, one obstacle is the limited experience with the use of NGS, specifically with the informed consent procedure, in the clinical setting. Moreover, little research has been done on the views of health professionals and patients 17.

Worldwide, several clinics have started to use NGS on a more routine basis. The Department of Clinical Genetics of the VU University Medical Center Amsterdam (VUMC) added exome sequencing to their range of genetic testing resources for diagnostics at the beginning of 2012. Patients whose previous clinical and genetic evaluation did not lead to an etiologic diagnosis are considered eligible for this approach. Patients invited for exome sequencing at the VUMC receive pre- and post-test counseling by a clinical geneticist and written informed consent is requested. Patients (or their representatives) consent to sequencing of the whole exome, with targeted analysis first, as well as the possibility of subsequently less targeted analysis of the data (if needed), including the chance of being informed about potential unsolicited findings (Fig. S1, Supporting information). Results clearly related to the phenotype of the patient are to be directly communicated to the clinical geneticist and subsequently to the patient. Results with potential clinical relevance but unrelated to the clinical enquiry are to be first discussed by an independent Advisory Board. This Board was introduced to prevent an internal conflict arising in the treating physician between the urge to follow up on the right of the patient not to know and the physician's duty to care. Although several models for ethical support have existed in different settings 18,19, the use of an independent Advisory Board for the legal, clinical and psychological circumstances of a specific case is (to our knowledge) a new concept in the clinical genetic setting 16. In the currently used informed consent procedure, as a pilot phase, the patient has no option to choose not to be informed about (thus to opt-out for) potential unsolicited findings. Where a patient indicates that she/he wishes not to be informed about unsolicited findings, she/he is considered ineligible for exome sequencing. Although there might be sound arguments to choose this procedure and similar procedures have been implemented in other University Medical Centers in the Netherlands, it is questionable whether it does justice to the patient's right to refuse to know about specific health information 16.

In this article we explore the first experiences and views of the stakeholders involved in the current procedure, including patients and professional experts, to gain insight into the needs for an optimal informed consent procedure for exome sequencing. The research questions were: (i) How do professional experts view the current informed consent procedure?; (ii) What are the experiences among the clinical geneticists and patients/legal representatives involved?

## Methods

The study consisted of two elements: (i) An interview-study with professional experts, and (ii) case-study observations with subsequent interviews.

The VU University Medical Ethics Committee approved the study protocol. All participants gave consent prior to the interview/observation.

### Interviews with professional experts

Semi-structured interviews were conducted with professional experts to explore their views on the needs for an optimal informed consent procedure for diagnostic exome sequencing, and to assess their opinion on the current procedure.

#### Participants in the interview study

Professional experts were selected based on their profession and expertise via the researchers' (national) network, and recruited through purposeful sampling with an invitation by phone or e-mail. The experts were affiliated to four of eight University Medical Centers in the Netherlands. All invited professionals participated in the study. Participants were: three clinical geneticists, three (clinical) molecular geneticists, two ethicists, one legal expert, one quality manager from a clinical genetics department, and two representatives of the Dutch Genetic Alliance (VSOP: The umbrella organization of national parent and patient organizations for genetic and/or congenital disorders), one of whom gave a written response due to time constraints.

#### Interview guide and procedure

An interview guide was developed in a multidisciplinary team including a molecular geneticist, clinical geneticist and health scientist. Topics addressed included: (i) reflection on the current informed consent procedure at VUMC, and (ii) advantages and disadvantages of an opt-out procedure for unsolicited findings.

Interviews lasted about half an hour and took place in the participants' work environment.

#### Analyses

The interviews were digitally recorded, transcribed verbatim, and full transcripts were member-checked and subsequently deidentified. Qualitative data indexing software (atlas.ti 5.2) was used for data coding. The transcripts were analyzed using thematic content-analytical techniques. Main codes were established for the core questions in the interview guide, while sub-codes were inductively formulated to identify emerging sub-themes. Two investigators (T. R. and C.v. A.) independently coded the first transcripts until consensus was reached on the code list. The main findings were discussed with other members of the study group.

### Case study: observation of patient–clinician interactions and interviews

Experiences of patients and genetic counselors with the informed consent procedure were studied through observation and semi-structured interviews.

#### Selection of cases for the study

All three patients eligible for exome sequencing for diagnostic purposes at the VUMC between March and June 2012 were included in the study. Cases were observed during the counseling. Two cases involved the testing of children and one case involved the testing of fetuses. The patient's legal representative and their clinician were subsequently interviewed about their experience.

#### Observation checklist, interview guide and procedure

During the counseling session extensive notes were taken by the researcher(s) (C.v. A. and/or T. R.). The observation checklist included: patient's body language, questions asked by the patient, and issues addressed by the clinician.

Topics addressed during the subsequent interview with the patients included: feelings during and after the session, intelligibility of given information, expectations of exome sequencing, and opinions about the communication of unsolicited findings.

The counselors were asked to reflect on the following topics in a short interview conducted the same day: transfer of information and the goal(s)of the counseling session, crucial points in the session, perceived complex issues, and points for improvement.

#### Data preparation and analyses

Depending on the preferences of the participants, the interviews were fully audio taped and transcribed and/or extensive notes were taken during the interview, and a summary was produced directly afterwards (two patient interviews were not recorded). All transcripts were deidentified and the audiotape was deleted after transcription. The notes and transcripts were analyzed by two researchers (T. R. and Cv. A.) by searching for relevant factors and associations with the research questions. These were coded and analyzed using atlas.ti 5.2. The quotes that follow have been translated from Dutch and were chosen to reflect a range of both consensual and dissenting views.

## Results

We identified six main themes regarding best practices for informed consent for NGS in diagnostics. The findings are summarized by theme and illustrated by quotes from professionals and/or findings from the case studies [see text boxes].

### Opt-out for unsolicited findings: needed but complex

During the interviews, many professionals expressed the need to give patients the possibility to opt-out of being informed about unsolicited findings, because patients have a right to refuse to know about specific health information:

A bottleneck [in the current procedure] is that people who don't want to know [unsolicited findings], don't have the option to do this [exome sequencing]. That's a problem which I hope we can solve in the future. (clinical molecular geneticist #1)

Patients should have a say; the doctor does not decide this for someone beforehand, what he should know or not, that to me is a good starting point. (ethicist #2)

Furthermore, it was mentioned that the introduction of an opt-out option for unsolicited findings would increase accessibility to NGS, as currently opting out of unsolicited findings would mean opting out of the whole test:

It [exome sequencing] should be available for everyone for whom it's clinically relevant. What you want to know or what you don't want to know about further results should not be relevant. (clinical molecular geneticist #2)

Disadvantages of an opt-out option for unsolicited findings involved the complexity of this decision and of the information provided prior to this:

I am personally not against an opt-out system, but an opt-out system only works if people are sufficiently informed, and as soon as they feel hesitance […] can opt out of it. (ethicist #1)

It was also mentioned that an opt-out for all possible unsolicited findings might be undesirable due to the duty to care of the physician:

To me […] it would seem very unpleasant when there is an unsolicited finding and someone has checked the box saying that he/she doesn't want to know anything about it. That you have this information, that you know it could be very important [for the patient]. To me, that would be a very undesirable situation. (clinical geneticist #3)

### Learning phase: need for more experience

Experiences with exome sequencing are new and therefore the current procedure might need adjustment over time; it was primarily seen as a learning process. Evaluating and documenting experiences with exome sequencing, the informed consent procedure and (the feedback of) unsolicited results was considered important. Additionally, although an opt-out for unsolicited findings was preferred by most respondents, more experience with exome sequencing was considered a prerequisite to make opting out a truly informed decision:

We are still in a learning phase [with exome sequencing]. Both for the medical specialist and for the lab specialist. Once things have become clearer it will be possible to define the options to choose from better [in an opt-out procedure]. (clinical molecular geneticist #3)

### Need for an Advisory Board for unsolicited findings?

When discussing the use of an Advisory Board for decisions on the actual communication of unsolicited findings to patients, respondents expressed both advantages and disadvantages. One of the main advantages mentioned was that, while leaving the main responsibility with the treating clinician, the Advisory Board could support the clinician in deciding what is best for a particular patient:

The question is whether one person should be responsible for that decision [whether to communicate an unsolicited finding to a patient]. It's better if more people are involved in that decision. (clinical molecular geneticist #1)

You are giving advice, the responsibility is always with the individual doctor or researcher, but I think it [Advisory Board] could be very supportive for the clinic. (legal expert #1)

Making use of an Advisory Board could lead to a more informed decision and the decisions would be more uniform amongst different clinicians.

Disadvantages of an Advisory Board mentioned were that it might undermine patients' autonomy. This was also expressed by patients themselves [see text box 1].

**Text box 1: An Advisory Board and personal autonomy: patient's perspective**

**Case 2 (Couple with multiple terminated pregnancies after ultrasound detection of multiple congenital anomalies in the fetuses at 20 weeks pregnancy)**

When the interviewer asks about the content of the counseling session, the father responds:

I also heard that someone makes that decision on the basis of ethics and that's what made me think: Well, aren't we man enough to decide for ourselves to decide what [unsolicited findings] we think is responsible to hear or not.

It was recognized that because involvement of the Advisory Board for every unsolicited result is time-consuming, it might be a temporary solution:

It is of course something that in the long run will be unmanageable. At a certain point in time, we will have to report our findings within two weeks and if every incidental finding has to pass a committee, that will never work. (clinical molecular geneticist #2)

### Context-dependent decision-making

Multiple respondents stressed that decisions on which unsolicited findings should be disclosed to patients are personal and context-dependent:

If you were to ask different people, […] whether they would like to know about certain information or not, the opinions would differ totally. That's also very personal, what people want. Even if you know a lot about it. That shows that it [deciding on disclosure of unsolicited findings] is something complex. (clinical molecular geneticist #1)

Differences in expectations and the personal weighing of potential benefits of a diagnosis with NGS against harms of potential unsolicited findings (proportionality) was also indicated in the case studies [see text box 2].

**Text box 2: Context-dependent decision-making: patient's perspective**

**Case 2 (Couple with multiple terminated pregnancies after ultrasound detection of multiple congenital anomalies in the fetuses at 20 weeks pregnancy)**

This couple decides not to give consent for exome sequencing. Although the mother would like to have a second child (they have one healthy toddler), the father is hesitant (partly due to his wife's age). Because of the unclear benefits of having a diagnosis of their previously unborn children, they feel it might not weigh up against the risk of unsolicited findings, as the father says:

I tend to lean towards: I don't want to know anything at all. I would only want to know if it is relevant with regard to…, but I do understand that that is not always possible.

**Case 3 (Parents of multiple children with dysmorphic features and developmental delay)**

A couple of which the woman is mildly cognitively impaired. Father is the legal representative of the family and speaks Dutch, although it is not his native language. Unsolicited findings do not seem of great importance to the father. He explicitly states:

The chance of knowing (the diagnosis) is more important than that (risk of unsolicited findings).

### Complexity of information provision

The information that is required for informed consent in this setting is complex, although the clinicians seemed confident in meeting the patient's needs. The concept of exome sequencing, possible unsolicited findings and comprehension of the possible consequences was perceived difficult to render [see text box 3]:

In any case I think that that it's very naïve to think that a patient is more able to choose [which results to receive] when he knows more. There are limits to what patients can comprehend. Decision-making in principle does not get easier, the more elaborately a patient is informed. It's definitely not about the quantity of information, but about the quality. The quality is important and also a discussion [with the patient]. (ethicist #2)

Can you really give informed consent when you look so widely [at the genome]? Is that manageable for patients? One can rightly question that. (patient representative Dutch Genetic Alliance #1)

**Text box 3: Complexity of information provision: patient's perspective**

**Case 1 (Couple with multiple children with congenital neurological phenotype without cognitive impairment)**

Father speaks Dutch fluently and mother understands Dutch but only speaks a little. During the counseling session it was explained to the parents that unsolicited results of the test in their children could also reveal relevant information for themselves (due to inheritance). When asked by the clinician how that made the father feel, his response showed he interpreted this message differently:

Of course it [the result] is of importance for me, because the care for my children is very important for me.

Although during the counseling session every sentence on the consent form was discussed with this father, the interview made clear that he had not understood what he had signed for or why:

I have often been asked to give permission for tests and I think this is very good. It makes me feel secure that other people cannot see what is being done.

### Division of responsibility between clinical geneticist and lab

Currently, patients give their consent beforehand for every analysis of the sequence that is needed to find a diagnosis. Several professionals expressed the need to decide on a more specific analytical strategy before a patient is asked for informed consent. This requires a new division of responsibility and close collaboration between the clinical geneticist and the clinical molecular geneticist:

Before patients come for counseling, there needs to be communication with me to sort out which [analytical] strategy to follow for this family. (clinical molecular geneticist #1)

I think that is going quite well now, I am quite happy about it […]. I think we have close contact with the people from the lab. By having those [regular clinical] meetings, but also personally I can call or e-mail them very easily or make an appointment. (clinical geneticist #3)

## Discussion

This study gives an impression of the first experiences of patients and clinicians, and presents the views of different professional experts on the informed consent procedure for NGS for diagnostic purposes. Whereas most respondents would prefer an opt-out option for unsolicited findings, they also indicated that more experience is needed before consensus can be reached on how to properly facilitate informed decision-making of patients. It has been previously argued that, in the context of NGS for diagnostic purposes, patients have a right to decide at least to a certain extent what testing to undergo and what information to receive 10,13. Although different models for information and choices for feedback of unsolicited findings have been developed 8,20, little empirical research has been conducted so far. Parties in the early adoption of exome sequencing in the Netherlands are well aware that they are still in a learning phase, especially with regard to managing expectations of patients and clinicians pertaining to unsolicited findings.

While ethical and practical disadvantages of an Advisory Board deciding on communicating unsolicited findings were mentioned, most respondents currently perceive the Board as providing important assistance to the clinician in deciding which unsolicited findings to disclose to patients. Some considered it not practically feasible or not an optimal solution. This might therefore be seen as a temporary solution until consensus can be reached on lists of genes in which pathogenic mutations should and should not be fed back to patients 16

The interviews and observations made clear that the possibility and potential consequences of unsolicited findings are difficult to comprehend. However, in two out of three cases in this study, parents of patients saw no harm in receiving unanticipated health information and seemed to be more focused on the possibility of receiving a clear diagnosis. Although previous studies describe that most patients are likely to choose to be informed about (all) unsolicited findings 21–23, it seems essential to continue paying attention to differences in expectations and the weighing of advantages and disadvantages (proportionality) between different patients in their own context 23. Given the complexity of the information provision, it remains unclear what exactly is needed to realize informed decision-making, in particular if people are not fluent in the language being spoken.

The results suggest that communication between the clinical geneticist and the clinical molecular geneticist is needed early in the process to assess the chances of a successful diagnosis and risks of (certain) unsolicited findings, as reported previously 16.

This study was conducted with a small sample size, with cases recruited from one center. Some of the findings may be context-specific and may not be representative for other settings. It is, however, one of the first studies on the views of different parties involved in the informed consent procedure. By using a mixed methods design and various perspectives the internal validity of the study increased. Awareness of being studied may have had some influence on the practice observed in the case studies. Although the results confirm to some extent what we had expected, we believe the findings could be of interest to other centers who are considering using NGS in diagnostics.

In conclusion, a close collaboration between the clinical molecular geneticist and the clinical geneticist seems essential to define the analytical approach that a patient is consenting to. Furthermore, there is a need for further empirical (follow-up) studies on the use of exome sequencing in diagnostics and patients' understanding and decision-making to be able to develop clear guidelines for the informed consent procedure. More experience with exome sequencing might teach us more about the frequency of unsolicited findings, which is essential information for patients in their decision process.

## Supporting Information

The following Supporting information is available for this article:

*Fig. S1.* Part of the consent form for exome sequencing in clinical diagnostics, VU University Medical Center Amsterdam, March 2012. Additional Supporting information may be found in the online version of this article.
