# Supplementary material for: Informed consent for exome sequencing in diagnostics: exploring first experiences and views of professionals and patients
Source: Clin Genet. 2013 Nov 4;85(5):417–22. doi: 10.1111/cge.12299 (PMC4231277; doi:10.1111/cge.12299)
Supplement: Supplementary file 1 — Fig. S1. Part of the consent form for exome sequencing in clinical diagnostics, VU University Medical Center Amsterdam, March 2012. Additional Supporting information may be found in the online version of this article. [file cge0085-0417-sd1.doc]

**Supporting information:**

1. I declare that I give permission to search my DNA/the DNA of the person for whom I am the legal representative, with exome sequencing for the following disorder:

………………………………………………………………………………………………………..

2. By signing this consent form I confirm I have been informed orally and in writing about exome sequencing and the possible outcomes of exome sequencing.

3. I agree with the following disclosure policy:

a. Every change or mutation in the DNA that is related, or could be, related to the initial enquiry for exome sequencing will be disclosed.

b. In the event that a change or mutation is found that is not related to the initial enquiry (an incidental finding), an independent committee of specialists will discuss whether the incidental finding is of direct relevance. Incidental findings that could be of direct relevance for my health and/or the health of my child will be discussed (the expectation is that the above will occur very rarely).

4. I understand that I may withdraw my consent at any time.

5. I confirm that my questions about exome sequencing have been answered to my satisfaction.

**Figure 1**: Part of the consent form for exome sequencing in clinical diagnostics, VU University Medical Center Amsterdam, March 2012.
